# Engaging HIV-HCV co-infected patients in HCV treatment: the roles played by the prescribing physician and patients' beliefs (ANRS CO13 HEPAVIH cohort, France)

**DOI:** 10.1186/1472-6963-12-59

**Published:** 2012-03-12

**Authors:** Dominique Salmon-Ceron, Julien Cohen, Maria Winnock, Perrine Roux, Firouze Bani Sadr, Eric Rosenthal, Isabelle Poizot Martin, Marc-Arthur Loko, Marion Mora, Philippe Sogni, Bruno Spire, François Dabis, Maria Patrizia Carrieri

**Affiliations:** 1 Unité de Maladies Infectieuses, Pôle Médecine, Hôpital COCHIN, Paris, France; 2 INSERM, U912 (SESSTIM), Marseille, France; 3 Université Aix Marseille, IRD, UMR-S912, Marseille, France; 4 ORS PACA, Observatoire Régional de la Santé Provence Alpes Côte d'Azur, Marseille, France; 5 Univ Bordeaux, ISPED, Centre INSERM U897-Epidemiologie-Biostatistique, F-33000 Bordeaux, France; 6 INSERM, ISPED, Centre INSERM U897-Epidemiologie-Biostatistique, F-33000 Bordeaux, France; 7 Substance Use Research Center, NYSPI, Columbia University, New York, NY, USA; 8 Hôpital Tenon-, INSERM U 707, Université Pierre et Marie Curie, Paris, France; 9 Hôpital de l'Archet, Université de Nice-Sophia Antipolis, Nice, France; 10 APHM hôpital Ste-Marguerite, Service d'Immuno-hématologie clinique, Marseille, France; 11 Institut Cochin, Université Paris-Descartes, INSERM U567-CNRS (UMR 8104), Paris, France; 12 APHP, Hôpital Cochin, Service d'Hépatologie, Paris, France

**Keywords:** HCV, HIV, Access to care, Alcohol, Primary care

## Abstract

**Background:**

Treatment for the hepatitis C virus (HCV) may be delayed significantly in HIV/HCV co-infected patients. Our study aims at identifying the correlates of access to HCV treatment in this population.

**Methods:**

We used 3-year follow-up data from the HEPAVIH ANRS-CO13 nationwide French cohort which enrolled patients living with HIV and HCV. We included pegylated interferon and ribavirin-naive patients (N = 600) at enrolment. Clinical/biological data were retrieved from medical records. Self-administered questionnaires were used for both physicians and their patients to collect data about experience and behaviors, respectively.

**Results:**

Median [IQR] follow-up was 12[12-24] months and 124 patients (20.7%) had started HCV treatment. After multiple adjustment including patients' negative beliefs about HCV treatment, those followed up by a general practitioner working in a hospital setting were more likely to receive HCV treatment (OR[95%CI]: 1.71 [1.06-2.75]). Patients followed by general practitioners also reported significantly higher levels of alcohol use, severe depressive symptoms and poor social conditions than those followed up by other physicians.

**Conclusions:**

Hospital-general practitioner networks can play a crucial role in engaging patients who are the most vulnerable and in reducing existing inequities in access to HCV care. Further operational research is needed to assess to what extent these models can be implemented in other settings and for patients who bear the burden of multiple co-morbidities.

## Background

Liver fibrosis progresses faster in HIV-HCV co-infected patients than among those with HCV alone [1]. While AIDS mortality has decreased sharply since the widespread introduction of antiretroviral treatment (ART) in 1996, end stage liver diseases now represent one of the leading causes of death in this population [2-5].

Treatment for HCV is available and cost-effective [6]; it cures 45% of patients with HCV genotype 1 infection and 75% of those with HCV genotype 2 or genotype 3 infection [7-9]. The current recommendations for the treatment of hepatitis C in mono-infected and HIV-HCV co-infected patients are much more liberal than before [10,11]. Previous indications which tended to delay or deny HCV treatment such as existing illicit drug/alcohol abuse, chronic renal disease, having undergone a liver transplant and severe psychiatric disease, no longer preclude patients from initiating treatment if adequate patient monitoring is ensured during follow-up. Rate estimates of treatment uptake in mono-infected HCV patients may vary considerably [12-19]. Most are obtained from clinic-based cohorts where rates range from 3% to 28% [12,20-23].

Patient characteristics, such as age, genotype, hepatic dysfunction, substance abuse, mental health issues and perception about treatment effectiveness and side effects [17,24] are important predictors of HCV treatment initiation. However, a significant proportion of variation in treatment uptake is explained by both structural factors (including those related to the experience of providers [19] and the model of care used to engage most excluded populations [25-27]) and patient social barriers, such as financial difficulties [14].

In HIV-HCV co-infected patients, HCV treatment uptake rates are usually lower than 50% and vary across the different regions of Europe [28,29]. In a large European survey published in 2006, the rate of treatment uptake was lower than 30%. In France, one cross-sectional study showed that 46% of HIV-HCV co-infected patients followed up in specialized centers for HIV care had received HCV treatment in 2004 [30] while other studies confirmed that barriers are found when engaging HIV-HCV co-infected individuals in HCV care [31,32].

In this population, several factors, not only those linked to HIV or HCV infections, might play a role in treatment uptake. It has been shown for example that isolation, lack of social support and legal issues are related to HCV treatment initiation [33] and that drug use and opioid dependence are conditions limiting not only access to liver biopsy [34,35] but also referral for [36,37] and initiation of HCV treatment [38]. It has also been consistently shown that opioid maintenance treatment facilitates HCV treatment initiation [39], especially in the case of one-site models of care.

Moreover, patient beliefs (such as fears of adverse effects of treatment or the conviction that treatment is ineffective) could also negatively influence uptake of HCV treatment.

It is worthwhile noting that HIV-HCV co-infection is a severe medical condition that requires a thorough evaluation of liver fibrosis and HIV status before starting HCV treatment. For these reasons HIV-HCV treatment is mainly initiated in hospitals. Though care for both diseases is free in France, some of the most vulnerable populations have less access to hospital services, and are more likely to attend primary care or non-referral clinics. However, in France, many non-specialized physicians, particularly those engaged in primary care for vulnerable populations like drug users or HIV-infected individuals, are members of or adhere to specialized networks. These networks are created solely on the initiative of local health care professionals engaged in improving access and quality of health care in a multidisciplinary manner. These networks may work in different ways and do not have a standardized structure unlike in other countries [25]; however there are some characteristics in common. They are all partially funded by the government; GPs belonging to a network do not work in the same hospital or health care setting but can divide their activity between their private office and hospital. Members of the network meet once per month to receive specific training and medical management update, including HIV and HCV clinical management.

In 2006, a large national cohort of HIV/HCV infected patients was implemented in France (ANRS CO13-HEPAVIH, N = 1040) and entailed the yearly collection of clinical and socio-behavioral data [40]. We used 3-year follow-up data from the HEPAVIH ANRS-CO13 nationwide French cohort to study the extent to which the characteristics of patients and physicians, may play a role in improving access to HCV treatment for HIV-HCV co-infected individuals.

## Methods

### Study design

In 2006, a nationwide prospective cohort study, ANRS CO 13 HEPAVIH, was initiated in 17 infectious diseases outpatient clinics delivering care to HIV-HCV co-infected patients in France.

Consecutive patients seen in 17 hospital wards between January 2006 and December 2008 and fulfilling the following criteria were enrolled in the cohort: aged 18 years or more, chronically infected with HIV and HCV as confirmed by a positive HIV antibody test and an HCV RNA assay (regardless of the clinical stage, gender and transmission group), and finally, provided written informed consent [40].

The study was approved by the Institutional Review Board of Cochin Hospital (Paris).

Clinical and biological data, including HIV RNA plasma viral load, CD4 cell count and the degree of liver fibrosis, together with data on HCV treatment initiation, were collected from a clinical research form completed by medical staff in outpatient hospital services. This form also contained information about HCV genotype, diagnosis of cirrhosis, Alanine AminoTransferase (ALT) level, Aspartate AminoTransferase (AST) level, HCV plasma viral load, previous HCV treatment, HIV antiretroviral treatment, and finally comorbidities (diabetes, hypertension, cardiovascular problems, renal dysfunction etc.)

Liver biopsy was performed at enrolment when possible. The results were documented and graded according to the Metavir system, measuring the activity of chronic hepatitis (none, minimal, moderate, severe) and the severity of fibrosis (none, portal fibrosis, portal fibrosis with rare septa, bridging fibrosis, cirrhosis). Whenever possible, a systematic assessment of liver fibrosis was also performed using two non-invasive methods: the first being Fibro Test™ and the second, elastometry by FibroScan™ Ultrasound examination was performed to screen for possible complications of liver disease. In the case of suspected or diagnosed cirrhosis, an endoscopic examination was prescribed. The schedule of follow-up visits was based on clinical practice as recommended by consensus conferences on hepatitis C, which are held every six months and every year for cirrhotic and for non-cirrhotic patients, respectively. Severe fibrosis (Metavir Score F ≥ 3) was assessed using an algorithm which took into account either liver biopsy, if performed less than one year before the visit or if not and the presence of indirect clinical signs of cirrhosis (oesophageal varices, ascites, liver encephalopathy or digestive bleeding) or results from non-invasive tests elastometry (Fibroscan^®^). The Fibroscan cut-off points used for Metavir score conversion were as follows: F0-F1: < 7.1 Kpa, F2: 7.1-9.5 Kpa, F3: 9.5-12.5 Kpa, F4: ≥ 12.5. Kpa [41].

### Patient and physician self-administered questionnaires

The patient self-administered questionnaire at baseline included items on socio-demographic characteristics (gender, age, having a secondary school certificate, having children, the desire for a child, living in a couple, employment and housing conditions), psychiatric disorders, addictive behaviors (depressive symptoms, alcohol consumption, cannabis, cocaine, heroin and tobacco use) and antidepressant treatment. It also included items on patient beliefs about the effectiveness and toxicity of HCV treatment, measured on a 4-point visual analogue scale.

A new variable was also built contrasting individuals who perceived treatment effectiveness as poor (score 1) and those who perceived treatment as highly toxic (score 4) with the rest of the study population.

Patient depressive symptoms were assessed using the Center for Epidemiological Studies Depression Questionnaire (CES-D) [42] which provides a global depression score ranging from 0 to 60, with gender-specific cut-off values (17 for men and 23 for women (18)). Score values above these cut-off points were taken as indicative of depressive symptoms. The 75% percentile of CES-D score (CES-D = 25) was also used to identify individuals with severe depressive symptoms.

A section of the self-administered questionnaire, based on the Symptoms Distress Module proposed by Justice et al. [43], also collected data about the occurrence of 39 treatment-related symptoms (defined here as self-reported side effects) over the previous four weeks, and the discomfort they caused. It was broadened to include questions on lipodystrophy symptoms. In the self-administered questionnaire, alcohol consumption was assessed by AUDIT-C [44,45] with three questions designed to compute the number of drinks per day. A standard drink containing 11-14 g of alcohol corresponds to one alcohol unit (AU). Women and men who reported drinking 2 and 3 AU or more per day respectively [46] were considered to have harmful alcohol consumption. Heroin, cocaine and cannabis use referred to the 4 weeks prior to the visit when the self-administered questionnaire was proposed.

Physicians who enrolled patients in the study and were the primary physician involved in HIV-HCV follow-up of patients included in the cohort were provided with a self-administered physician questionnaire at the enrolment visit and at scheduled annual visits. This questionnaire collected data about patient socio-behavioral characteristics, physicians' experience with and perceptions of their patients as well as information about their patients concerning depression, alcohol use and history of suicide attempts. Two structural hospital-specific variables were built: the number of patients enrolled in the cohort in each hospital and the proportion of patients in each center followed up by a general practitioner.

### Outcome and study subjects

The outcome variable was the initiation of HCV treatment, defined as the first prescription of pegylated interferon and ribavirin, during the first three years of the cohort follow-up.

In order to study the impact of physician and patient clinical and socio-behavioral characteristics at enrolment associated with initiation of HCV treatment, the analysis was restricted to those patients having chronic hepatitis C (defined by having a positive HCV RNA), who were peg-IFN + Ribavirine naive at enrolment in the cohort. Among this population, individuals with decompensated cirrhosis or hepatocellular carcinoma who had undergone transplantation were excluded. In addition, patients who had not completed any part of the self-administered questionnaire at enrolment were also excluded.

### Statistical methods

A multiple imputation approach was used for variables presenting less than 10% missing data to obtain more precise estimates. We used the Multivariate Imputation by Chained Equations method [47] with m = 5 multiple imputations, and with logistic regression and predictive mean matching imputation models respectively for binary and continuous variables. Scores (for example the CES-D score) were recalculated after the imputation step.

Clinical and biological data, as well as data from the patient self-administered questionnaires, were compared between patients who had already started HCV treatment during the first three years of the cohort follow-up and those who had not. T-tests, Mann-Whitney, χ^2 ^or Fisher tests were used, when appropriate, to compare patient characteristics. Odds ratios (OR) and their 95% confidence intervals were calculated to quantify the strength of association between the outcome and the correlates. As the duration of patient follow-up was short and enrolment characteristics were tested as predictors of subsequent initiation of HCV treatment, a logistic regression model was performed to determine factors associated with initiation of HCV treatment. Variables with a liberal p value < 0.20 in the univariate analysis were considered eligible for the multivariate model which was built using a backward procedure based on the log-likelihood ratio test. All the analyses were performed using Stata 10.0.

Mixed logistic regression models were also used to verify whether the specialty of the physicians was associated with specific characteristics of patients while accounting for inter-variability between hospital centers, these latter being considered as a random effect.

## Results

### Selection of the study sample

From the initial data set (N = 1040 of patients with chronic hepatic C), 779 patients were peg-IFN and ribavirin naive. The following patients were not included in analyses: individuals with decompensated cirrhosis (N = 14), those who had undergone a liver transplantation (N = 1) or who had hepatocellular carcinoma (N = 2) and those who had not completed any part of the self-administered questionnaire at enrolment (N = 162). In the end, 600 individuals were eligible for the present analysis.

No significant differences were found in terms of gender, age, CD4, HIV viral load and HCV viral load, ASAT, ALAT, HCV genotype and fibrosis at baseline, whether follow-up by a general practitioner or not, between those who filled in the self-administered questionnaires (N = 600) and those who did not (N = 162).

### Characteristics of the study sample

**Figure 1:**
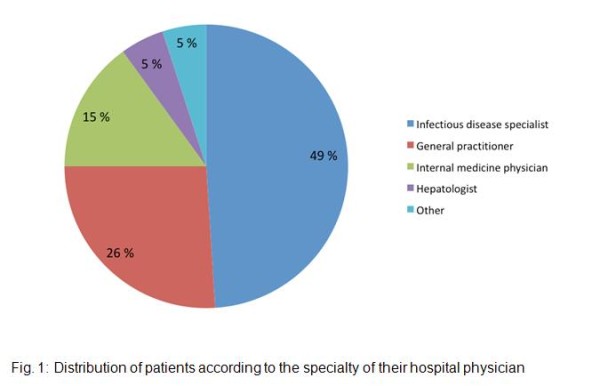
**Distribution of patients according to the specialty of their hospital physician**.

Table 1 lists the characteristics of the study sample (N = 600). Median [IQR] age was 44[41-47] years, men accounted for 68% and most patients (63%) were HIV-infected through injecting drug use (IDU). At enrolment 59% of the patients had undetectable plasma HIV RNA and median CD4 cell count/mm^3 ^was 441[295-650]. Twenty percent had HCV genotype 2 or 3 while 67% had genotype 1 or 4. Among the study sample, 21% presented with liver cirrhosis at enrolment and 12% had a CD4 count < 200/mm^3^. Nine percent had already been exposed to a HCV treatment different from PEG-IFN + ribavirin. Most patients (89%) were receiving ART at enrolment in the cohort. The median [IQR] number of self-reported side effects and self-reported side effects causing discomfort were 7[2-14] and 2[0-7] respectively. Among the study population, 49% of the study patients were mainly followed up by an infectious disease specialist, 26% by a general practitioner, 15% by an internal medicine physician, 5% by hepatologists and the remaining 5% by other specialists (Figure 1).

**Table 1:** Characteristics of patients (N = 600) according to HCV treatment initiation during follow-up (ANRS CO13 HEPAVIH cohort.)

	HCV treatment initiation			
	**NO** **% or median** **[IQR]** **N = 476**	**Yes** **% or median** **[IQR]** **N = 124**	**Total** **sample** **% or median** **[IQR]** **N = 600**	**p-value§**

Female gender	34	27	33	0.12
Age	44 [41-48]	43 [41-47]	44 [41-47]	0.06
Born outside France	19	20	19	0.79
HIV transmission group				0.51
IV drug use	63	64	63	
Heterosexual	16	12	15	
Homosexual	10	14	11	
Other or unknown	11	10	10	
HCV genotype				0.48
1 or 4	66	72	67	
2 or 3	18	25	20	
5	1	0	1	
Unknown	15	2	13	
ASAT	47 [34-71]	52 [37-94]	48 [35-74]	0.06
ALAT	52 [35-84]	65 [42-91]	56 [35-87]	0.04
Previous exposure to IFN (not PEG) + ribavirine	9	9	9	0.95
Severe fibrosis (Metavir F3 or F4)	22	45	27	< 10^-3^
Fibrosis score				
F0-F1	59	40	55	< 10^-3^
F2	18	16	18	
F3	7	17	9	
F4	15	28	18	
CD4 < 200/mm^3^	12	11	12	0.75
Undetectable HIV viral load	62	60	81	0.69
Antidepressants use	20	19	20	0.93
Harmful alcohol consumption^a^	11	15	12	0.25
Daily cannabis use	15	18	16	0.52
Cocaine use	8	10	9	0.59
Heroin use	2	4	3	0.47
More than 20 cigarettes consumed per day	32	33	32	0.88
Depressive symptoms^b^	40	41	40	0.80
Number of self-reported symptoms	7 [2-14]	6 [2-14]	7 [2-14]	0.53
Receiving ART	89	92	89	0.32
Number of years since first HIV positive test	17 [13-20]	16 [13-20]	17 [13-20]	0.11
Number of years since first HCV positive test	11 [7-15]	9 [4-13]	10 [6-15]	0.004
Patients negative beliefs HCV treatments^c^	42	29	39	0.03
Patient- high adherence as perceived by the physician	38	47	40	0.10
Patient- alcohol problems as perceived by the physician	20	21	20	0.72
Patient- suicide attempts as reported by the physician	8	8	8	0.99
Patient- history of multiple treatments for depression as reported by the physician	19	20	19	0.77
Followed up by a general practitioner	22	39	26	0.001
Liver biopsy at enrolment	32	52	36	< 10^-3^

^a ^Defined as the consumption of more than 90 alcohol units/month for men and 60 alcohol units/month for women

^b ^Patients were defined as having depressive symptoms if CES-D > 17 for men and > 23 for women

^c ^Beliefs were measured on a 1-4 visual analogue scale. Believes HCV treatment to be non-effective (score 1) and associated with large number of side effects (score 4)

§ A Chi-squared and T-test was performed for qualitative and quantitative variables respectively

The median [IQR] percentage of patients followed up by general practitioners was 6% [0%-35%] while the median [IQR] number of patients enrolled in each hospital center was 27[15- 60].

Among the 600 patients followed up for a median of 12 months [12-24], 124 (20.7%) started HCV treatment during follow-up, corresponding to an HCV treatment incidence of 15 new events per 100 person-years. The proportion of severe liver fibrosis was respectively 45% and 22% for those initiating HCV treatment or not (p < 0.01, Table 1 Evaluation of liver fibrosis at enrolment (+ - one year), with liver biopsy, FibroTest™ or FibroScan™ was available for 209, 60 and 315 patients respectively. Among patients classified as F3-F4, 40% of those diagnosed by liver biopsy and 24% of those diagnosed with non invasive methods (p < 10-3). started HCV treatment during follow-up.

Table 2 shows the results of the analyses identifying factors associated with HCV treatment initiation. In the univariate analyses, patients were more likely to receive treatment if they had severe fibrosis (OR [95%CI]: 2.66[1.74-4.09]) and had had liver biopsy (OR [95% CI]: 2.31 [1.54-3.45]). Individuals who initiated HCV treatment were more likely to be men, to be younger, to have no children and to have been HIV or HCV diagnosed more recently. Individuals followed-up by a general practitioner working in hospital outpatient services were more likely to receive HCV treatment whereas those reporting negative perceptions about HCV treatment effectiveness and toxicity initiated HCV treatment significantly less frequently. In this study population, HIV immuno-suppression and virological status were not found to be associated with treatment initiation. After adjustment for age (AOR [95%CI]: 0.69[0.48-0.98]), number of years since HCV diagnosis (AOR [95%CI]: 0.69[0.49-0.97]), having had a liver biopsy (AOR [95%CI]: 2.20[1.44-3.35]), both severe fibrosis (AOR [95%CI]: 2.87[1.83-4.51]) and being followed up in hospital by a general practitioner (AOR [95%CI]: 1.71[1.06-2.75]) remained significantly associated with an increased likelihood of HCV treatment initiation. By contrast, patients with negative beliefs about both HCV treatment effectiveness and toxicity were less likely to receive HCV treatment (AOR [95%CI]: 0.58 [0.37-0.92]).

**Table 2:** Factors associated with HCV treatment initiation during the first three years of cohort follow-up; Univariate and multivariate analyses based on a logistic regression model (n = 600, ANRS CO13 HEPAVIH cohort.)

	Univariate analysis		Multivariate analysis	
	p-value	OR (95% CI)	p-value	AOR (95% CI)
Female gender^§^	**0.12**	**0.70 (0.45-1.09)**		
Age^a§^	**0.06**	**0.73 (0.52-1.01)**	**0.04**	**0.69 (0.48-0.98)**
Born outside France^§^	0.81	1.06 (0.64-1.76)		
Secondary school certificate^§^	0.84	1.04 (0.68-1.60)		
Having children^§^	**0.05**	**0.63 (0.40-0.99)**		
Living in a couple^§^	0.91	1.02 (0.69-1.52)		
Steady partner^§^	0.57	1.13 (0.75-1.70)		
Employment^§^	0.85	1.04 (0.70-1.54)		
Owner or renter of their house^§^	0.24	0.68 (0.36-1.28)		
Good housing conditions^b§^	0.53	1.19 (0.70-2.02)		
Treated several times for depression^§^	0.97	0.99 (0.59-1.66)		
Antidepressants use^§^	0.93	0.98 (0.59-1.61)		
Harmful alcohol consumption^c§^	0.25	1.40 (0.79-2.50)		
Daily cannabis use^§§^	0.57	1.18 (0.67-2.06)		
Cocaine use^§§^	0.75	1.12 (0.55-2.27)		
Heroin use^§§^	0.54	1.45 (0.44-4.77)		
More than 20 cigarettes consumed a day^§§^	0.88	1.04 (0.67-1.59)		
Depressive symptoms^d§^	0.96	1.01 (0.65-1.56)		
Number of self-reported symptoms^§§^	0.53	0.99 (0.97-1.02)		
Receiving ART^§^	0.32	1.43 (0.70-2.89)		
Number of years since first HIV positive test^a^	**0.10**	**0.75 (0.53-1.06)**		
Number of years since first HCV positive test^a§§^	**0.01**	**0.64 (0.47-0.88)**	**0.03**	**0.69 (0.49-0.97)**
Patients negative perceptions about HCV treatment^§§e^	**0.03**	**0.60 (0.38-0.94)**	**0.02**	**0.58 (0.37-0.92)**
Severe fibrosis^§f^	**< 10^-3^**	**2.66 (1.74-4.09)**	**< 10^-3^**	**2.87 (1.83-4.51)**
CD4 cell count < 200^§§^	0.89	0.95 (0.48-1.89)		
Undetectable HIV viral load^§§^	0.69	1.09 (0.72-1.65)		
Patient high adherence as perceived by the physician^§§^	0.21	1.30 (0.87-1.94)		
Followed up by a general practitioner^§§^	**0.01**	**1.93 (1.20-3.12)**	**0.03**	**1.71 (1.06-2.75)**
Patient alcohol problems as reported by the physician^§§^	0.75	0.92 (0.53-1.57)		
Patient suicide attempts as reported by the physician^§§^	0.99	1.01 (0.44-2.28)		
Patient history of multiple treatments for depression as reported by the physician^§§^	0.77	1.09 (0.62-1.90)		
**Liver biopsy at enrolment**	**< 10^-3^**	**2.31 (1.54-3.45)**	**< 10^-3^**	**2.20 (1.44-3.35)**

^a ^Per ten year increase

^b ^Good housing conditions were defined as ranks 3 and 4 (quite or very comfortable vs. uncomfortable or quite uncomfortable) using a four-point Likert scale

^c ^Consumption of more than 90 alcohol units/month for men and 60 alcohol units/month for women

^d ^Patients were defined as having depressive symptoms if CES-D > 17 for men and > 23 for women.

^e ^Beliefs were measured on a 1-4 visual analogue scale. Believes HCV treatment to be non-effective (score 1) and associated with large number of side effects (score 4)

^f ^Severe fibrosis was defined according to Metavir score F3-F4 vs. F0-F1-F2.

^§ ^< 5% of missing values estimated using multiple imputation

^§§ ^< 10% of missing values estimated using multiple imputation

In the final model, when the variable "followed up by a general practitioner" was replaced with the aggregated variable "proportion of patients followed up by a medical practitioner" in each center (AOR [95%CI]: 1.13 (1.04-1.23) per 10% increase) and the number of patients enrolled in the cohort in each center (AOR [95%CI]: 1.10 (1.02-1.18) per 10 patient increase), both variables remained significantly associated with HCV treatment initiation.

It is interesting to note that with respect to the other patients, those receiving care in hospital from a general practitioner working in an infectious diseases outpatient clinic were more likely to present with severe liver fibrosis (37% vs. 23%, p = 0.003) and harmful alcohol consumption (16% vs.8%, p = 0.007). Fewer were employed (38% vs. 52%, p = 0.007). Furthermore, they were also more likely to present with severe depressive symptoms (30% vs. 22%, p = 0.05). These comparisons remained significant even after controlling for inter-center variability using a mixed logistic regression model for each outcome (liver fibrosis, harmful alcohol consumption, employment, severe depressive symptoms).

## Discussion

The present study conducted in a large cohort of HIV-HCV infected patients enrolled and followed up in France shows that barriers to HCV treatment do not solely depend on patient characteristics and physicians' perceptions of their patients. It highlights that patient beliefs about HCV treatment can also significantly delay HCV treatment initiation. The results suggest that general practitioners working in hospitals play a major role in engaging patients in HCV care. As expected and in accordance with current guidelines for HCV treatment prescription, we found that having a liver biopsy and presenting with severe fibrosis remain major clinical determinants of HCV treatment initiation. It is worthwhile noting that the study started when non-invasive procedures had just become available and their sensitivity to detect advanced liver disease in co-infected was not completely known. It is likely that the association found with having had a liver biopsy reflects common hospital practices at the beginning of the cohort, so this adjustment enables us to control for differential access to liver biopsy.

These results remained valid after adjustment for age and time since HCV diagnosis, which were both inversely correlated with initiation of treatment.

Patients followed up by general practitioners working in infectious diseases outpatient clinics were characterized by more severe liver fibrosis and reported significantly higher levels of alcohol use than their counterparts followed up by specialists. The former were more likely to receive HCV treatment than the latter. This positive effect may be the result of an awareness campaign for HCV treatment regularly conducted by general practitioner-hospital networks. It could also be the effect of a national campaign to fight HCV, conducted among general practitioners in France between 1999 and 2002 [48]. Furthermore, there is a considerable number of general practitioners involved in specialized care networks for drug users. These same physicians are often in charge, on a part-time basis, of following up drug users in hospitals, and consequently provide more comprehensive care for drug dependence, HIV and HCV. It is also possible that the physicians in our study had already met this group of patients with advanced HCV disease and alcohol abuse in their private office and subsequently convinced them to receive appropriate clinical assessment and treatment for their HCV infection in the hospital outpatient clinics where they worked as general practitioners. The fact that such patients had severe fibrosis more frequently than others confirms that their hospital based follow-up started when HCV treatment was needed. Therefore, thanks to the link between hospital services and general practitioners, hidden populations in France can be successfully engaged in HCV care. In other settings, the creation of ad-hoc multidisciplinary networks to engage patients in HCV treatment has also been found to be successful not only in terms of access [25] but also in terms of response to HCV treatment [26].

Qualitative research would be required to further investigate the above hypotheses, as the survey instruments and study design used here are limited.

This is the first quantitative study of coinfected HIV-HCV individuals to highlight the association between patients' reduced access to HCV treatment and their fear of both its ineffectiveness and side effects.

It is difficult to say to what extent such beliefs may have been influenced by the information provided to patients by their physicians, or by friends' or relatives' own experiences with HCV treatment. One survey conducted among French drug users has already underlined the negative perception which drug users have about HCV treatment effectiveness and toxicity [24]. Other qualitative and quantitative studies outside France have confirmed the negative impact of opioid dependence and/or negative perceptions about treatment on engaging in HCV treatment [39,48-50]. In our study however no significant association was found between reporting such beliefs and opioid dependence. An association between patients' reluctance and engagement in HCV treatment was found in another study [51] where 2 in 3 patients declined HCV treatment. It also confirms that HCV treatment uptake is probably lower in HIV-HCV co-infected patients, something already demonstrated in previous research [52].

As the main concern of physicians involved in the care of HIV-HCV co-infected patients is to maintain high levels of adherence to ART, they may be reluctant to add HCV treatment to ART due to the fear that side-effects and depressive symptoms associated with the former [53,54] might compromise the effectiveness of the latter. Nevertheless, recent data from the same cohort have highlighted that adherence to ART is enhanced by engagement in HCV treatment [55]. There is evidence today to show that while individuals needing HCV treatment often report not having enough information about both its associated risks and benefits [17], they tend to show much greater interest in commencing treatment once informed [56]. Whatever the reason for this, our findings underline the need to reduce barriers to HCV treatment in this population by improving both the patient-provider relationship and communication about virological failure, side effects and their management during HCV treatment.

As new anti-HCV drugs with increased efficacy and better tolerance will soon be available, it is possible that the fear of side-effects in this population may be attenuated and that initiation of HCV treatment will increase in HIV-HCV co-infected individuals.

Unlike what has been reported in the literature, we did not find any significant association between HCV treatment initiation and the following factors: physicians' beliefs about patients' adherence, alcohol abuse or opioid dependence, patient self report of alcohol or drug use [57]. This lack of association is probably attributable to the more liberal guidelines available today regarding access to HCV treatment. Although these same guidelines suggest that a patient have a "stabilized" lifestyle before starting HCV treatment, they do not exclude drug users or alcohol abusers from HCV treatment [11]. Nevertheless, alcohol abuse remains a major barrier to treatment [58]. The lack of association between opioid dependence and delay in HCV treatment in our study may be related to changing perceptions by French HIV physicians as they increasingly realize that opioid dependence is not a limiting condition for sustained adherence, even for life-long treatments like antiretrovirals [59].

Finally, factors directly related to HIV disease (such as immune restoration) have also been shown to impact patient HCV treatment uptake [28]. However, such associations were not found in our study, possibly due to the fact that most of our patients were receiving ART and had good immune restoration.

Some limitations of the study need to be acknowledged. Data on addictive behaviors were based on self-reports, whose use is often questioned due to possible social desirability bias. Nevertheless, the validity and reliability of self-reports about substance use have been established in many studies using similar methods for data collection about addictive behaviors [60]. Moreover, in France, the health insurance system allows even marginalized populations to have free access to care [61], not only for HIV but also for drug dependence. That is why we can presume that HIV-HCV co-infected patients (including drug users) are adequately represented in the sample.

The validity of the results is restricted to hospital-based settings, yet many patients may be engaged in HCV care in other settings (primary care, opioid substitution treatment etc...).

In this analysis we were unable to account for specific characteristics related to the model of HCV care used in each hospital but it is important to point out that all hospital centers were academic, urban and multidisciplinary. While another limitation could be the use of various enrolment data as potential correlates of HCV treatment initiation instead of time-varying factors, the duration of the follow-up was so limited that it is unlikely that the correlates found could significantly change over time.

It is also possible that the exclusion of patients already treated (i.e. patients who were treated before follow-up), may have hidden associations with other medical specialties among prescribing physicians. It is possible that we focused on a selected population which has not yet been treated for two main reasons: 1) not yet eligible for HCV treatment according to current recommendations or 2) because HCV treatment has been previously delayed due to negative perception about their possible treatment adherence or readiness to start. This could potentially limit the external validity of the results though we think that this study group reflects the reality of patients currently needing treatment in countries with similar guidelines and contexts.

## Conclusions

Initiation of HCV treatment in France is related not only to clinical characteristics, for example the severity of liver fibrosis, but also to patients' perceptions about the effectiveness and constraints of this treatment and to their relationship with their physicians. Hospital-primary care networks can play a crucial role in engaging the most vulnerable patients and reducing inequities in access to HCV care. Further operational research is needed to assess to what extent these models can be implemented in other settings and for patients who, like HIV-HCV co-infected patients, bear the burden of living with multiple co-morbidities.

## Competing interests

DSC, JC, MW, FBS, MAL, PR, MM, FD, BS, and MPC has no competing interests to declare. PS was invited at Board, workshop participations or meeting of Gilead, Bristol-Myers Squibb, Schering-Plough/MSD, Roche, Janssen. He was Sub-investigator in HCV trials: Bristol-Myers Squibb, Roche, Schering-Plough/MSD, Boehringer Ingelheim, Tibotec, Vertex, Janssen IPM was invited at Board, workshop participations or meeting invitations: Gilead, Bristol-Myers Squibb, Schering-Plough/MSD, Roche, Janssen, Viiv health care, Abbott, Boehringer Ingelheim She was sub-investigator in HIV and HCV trials: Bristol-Myers Squibb, Roche, Schering-Plough/MSD, Boehringer Ingelheim, Tibotec, Gilead, Viiv Health care, Abbott ER received honoraria from Roche and Schering-Plough.

## Authors' contributions

DSC is the main investigator of the cohort, wrote the first draft of the introduction and of the discussion of the manuscript. JC performed the statistical analysis, wrote the methods and the results of the first draft of the manuscript and followed the different revisions of the manuscript before its acceptance. MW and MAL contributed to the methods section and revised the whole manuscript. PR, FD, FBS, PS, BS, MM contributed to the different revisions of the whole manuscript. ER, IPM were investigators of the study and contributed to the discussion of the results. MPC coordinated the data analysis strategy, contributed to the introduction and the discussion, revised the final manuscript and followed the different revisions of the manuscript before its acceptance. All authors read and approved the final manuscript.

## Pre-publication history

The pre-publication history for this paper can be accessed here:

http://www.biomedcentral.com/1472-6963/12/59/prepub
